# The Efficacy of Conversational AI in Rectifying the Theory-of-Mind and Autonomy Biases: Comparative Analysis

**DOI:** 10.2196/64396

**Published:** 2025-02-07

**Authors:** Marcin Rządeczka, Anna Sterna, Julia Stolińska, Paulina Kaczyńska, Marcin Moskalewicz

**Affiliations:** 1 IDEAS NCBR Warsaw Poland; 2 Institute of Philosophy Maria Curie-Skłodowska University Lublin Poland; 3 Philosophy of Mental Health Unit Department of Social Sciences and the Humanities Poznan University of Medical Sciences Poznań Poland; 4 Faculty of Mathematics, Informatics, and Mechanics University of Warsaw Warsaw Poland

**Keywords:** cognitive bias, conversational artificial intelligence, artificial intelligence, AI, chatbots, digital mental health, bias rectification, affect recognition

## Abstract

**Background:**

The increasing deployment of conversational artificial intelligence (AI) in mental health interventions necessitates an evaluation of their efficacy in rectifying cognitive biases and recognizing affect in human-AI interactions. These biases are particularly relevant in mental health contexts as they can exacerbate conditions such as depression and anxiety by reinforcing maladaptive thought patterns or unrealistic expectations in human-AI interactions.

**Objective:**

This study aimed to assess the effectiveness of therapeutic chatbots (Wysa and Youper) versus general-purpose language models (GPT-3.5, GPT-4, and Gemini Pro) in identifying and rectifying cognitive biases and recognizing affect in user interactions.

**Methods:**

This study used constructed case scenarios simulating typical user-bot interactions to examine how effectively chatbots address selected cognitive biases. The cognitive biases assessed included theory-of-mind biases (anthropomorphism, overtrust, and attribution) and autonomy biases (illusion of control, fundamental attribution error, and just-world hypothesis). Each chatbot response was evaluated based on accuracy, therapeutic quality, and adherence to cognitive behavioral therapy principles using an ordinal scale to ensure consistency in scoring. To enhance reliability, responses underwent a double review process by 2 cognitive scientists, followed by a secondary review by a clinical psychologist specializing in cognitive behavioral therapy, ensuring a robust assessment across interdisciplinary perspectives.

**Results:**

This study revealed that general-purpose chatbots outperformed therapeutic chatbots in rectifying cognitive biases, particularly in overtrust bias, fundamental attribution error, and just-world hypothesis. GPT-4 achieved the highest scores across all biases, whereas the therapeutic bot Wysa scored the lowest. Notably, general-purpose bots showed more consistent accuracy and adaptability in recognizing and addressing bias-related cues across different contexts, suggesting a broader flexibility in handling complex cognitive patterns. In addition, in affect recognition tasks, general-purpose chatbots not only excelled but also demonstrated quicker adaptation to subtle emotional nuances, outperforming therapeutic bots in 67% (4/6) of the tested biases.

**Conclusions:**

This study shows that, while therapeutic chatbots hold promise for mental health support and cognitive bias intervention, their current capabilities are limited. Addressing cognitive biases in AI-human interactions requires systems that can both rectify and analyze biases as integral to human cognition, promoting precision and simulating empathy. The findings reveal the need for improved simulated emotional intelligence in chatbot design to provide adaptive, personalized responses that reduce overreliance and encourage independent coping skills. Future research should focus on enhancing affective response mechanisms and addressing ethical concerns such as bias mitigation and data privacy to ensure safe, effective AI-based mental health support.

## Introduction

### The Potential and Pitfalls of Therapeutic Chatbots

Given the rapid development of advanced artificial intelligence (AI) assistants, ethics are required to move beyond focusing on isolated metrics such as model properties and outputs and aim at more holistically understanding their interaction with humans in real contexts [1]. Similarly, the growing popularity of conversational user interfaces has driven human-computer interaction (HCI) research focused on specific approaches to the research, design, and implementation of these interfaces. However, this research remains largely fragmented and lacks a unified approach to theory, methods, and design [2]. The exact role that conversational AI, or chatbots for short, can play in the realm of digital mental health is definitely debatable, as calling them digital therapists seems too far-fetched due to their still limited capabilities. Obviously, chatbots lack therapeutic autonomy as they are technological cognitive-affective artifacts able to influence users’ beliefs and emotional states but without many contextual clues essential for traditional therapeutic interventions [3]. Interactions with chatbots are disembodied, thus creating a different environmental niche than that of traditional therapy.

Therapeutic bots, which constitute software that usually uses some natural language processing and machine learning technologies, mark a transformative stride in mental health support. By simulating conversation and offering guidance, these bots aim to guide individuals through their cognitive biases and even mitigate some affect variability, providing immediate, easily accessible, and sometimes anonymized interactions. The main strength of therapeutic bots lies in their availability and consistency. They offer round-the-clock support, reaching individuals in remote or underserved areas where human therapists might not be accessible [4]. In addition, for some users (eg, individuals with autism), interacting with a bot alleviates the stigma or discomfort associated with seeking mental health support, fostering a sense of safety in expressing feelings and thoughts [5]. Similarly, there have been some theoretical claims (requiring further empirical validation) that chatbots may help some individuals with borderline personality disorder increase narrative coherence between therapeutic sessions, nurturing more effective patient-therapist communication [6]. However, the reliance on therapeutic bots introduces numerous challenges, primarily in the nuanced understanding of human emotions and the complex dynamics of second-wave cognitive behavioral therapy (CBT). Obviously, bots lack the genuine empathy, intuition, and depth of understanding that human therapists provide, potentially oversimplifying or misunderstanding overly complex emotional issues. Moreover, there exists a serious risk of overreliance on these bots in people with preexisting mental health conditions, with individuals potentially substituting professional human interaction for digital conversations, which might not always be equipped to handle severe or acute mental health crises. However, excessive panic about AI dependence in the general population seems to be rather ungrounded as there is little evidence to support that claim [7].

### Why Should Chatbots Address Human Cognitive Biases?

Addressing human cognitive biases through mental health chatbots is pivotal for several reasons, particularly given the expansive role they play in shaping human cognition, emotions, and decision-making. These biases, such as the tendency to anthropomorphize, overtrust, attribute emotional states, focus on illusory control over external events, or distort reality by losing the balance between the role of direct human actions and external factors in a given behavior, can exacerbate or contribute to mental health issues such as anxiety, depression, and low self-esteem. By identifying and rectifying these biases, therapeutic bots can possibly guide individuals toward healthier thinking patterns, promoting emotional well-being and resilience.

First, mental health chatbots that can accurately identify and address cognitive biases have the potential to provide immediate corrective feedback. This is crucial in a therapeutic context where timely intervention can prevent negative thought spirals, offering users a chance to reframe their thoughts in a more positive or realistic light. For instance, a bot that recognizes and challenges an individual’s tendency to engage in *all-or-nothing* thinking can help break cycles of negative thinking that contribute to depressive symptoms [8].

Second, integrating cognitive bias correction into chatbots democratizes access to some forms of CBT-based cognitive restructuring, making them available to a broader audience. CBT is a highly effective treatment for various mental health conditions, but access to it can be limited due to cost, availability of therapists, or stigma associated with seeking therapy. Therapeutic bots equipped to address cognitive biases can provide a form of CBT, making some of the core benefits of this therapy accessible to individuals who might not otherwise seek or receive it.

### Balancing Efficacy and Ethics

However, addressing cognitive biases through chatbots also presents significant challenges, including ensuring the accuracy of bias identification, the ethical use of collected data, and the need for bots to navigate the complex nuances of human psychology respectfully and effectively. Balancing these considerations is essential for developing the full potential of therapeutic bots in mental health support.

One advantage of therapeutic chatbots is that they might be preferred over human counselors by people who either have trouble with interpersonal communication (eg, people in the autism spectrum) or are more inclined to use them due to their condition (eg, mild to moderate anxiety or obsessive-compulsive disorder). For example, people in the autism spectrum are generally rather willing to disclose their diagnosis to the chatbot and ask for a tailored response [9]. However, this kind of tendency to choose chatbots over human counselors may come at a price. While sometimes facilitating more effective communication and possibly a form of web-based therapeutic alliance, an overtrust in AI-based therapeutic tools by users is also a possible source of harmful side effects when theoretically beneficial biases are unproportionally reinforced. It is the reason why enhancing affect recognition and minimization of bias reinforcement in chatbot design is crucial to ensure their safe and effective use among vulnerable groups.

One primary ethical issue to consider is the unintentional perpetuation of biases embedded in AI-driven chatbots, which can lead to skewed therapeutic recommendations or responses. These biases often originate in the training data, which may reflect societal prejudices, stereotypes, or historical inequities. Consequently, language models may exhibit biases in ways that could be detrimental to vulnerable populations, such as individuals with specific mental health conditions. For instance, biased responses related to gender, race, or socioeconomic status can influence a chatbot’s affective feedback, potentially altering therapeutic outcomes and undermining the model’s intended support function. One way to counteract those challenges is to consider rigorous methods to audit and mitigate biases, such as implementing fairness constraints during training and incorporating diverse datasets that better reflect the nuances of real-world interactions [10].

Privacy concerns are also paramount, especially in mental health applications in which sensitive personal data may be disclosed by users. The confidential nature of therapeutic conversations requires robust data protection to prevent unauthorized access or misuse. Although some chatbots are designed to handle conversations without storing individual session data, the risk remains that data could be accessed, hacked, or improperly managed, potentially exposing users’ mental health information. This issue is further complicated by the possibility that third-party developers or service providers may lack stringent data management protocols, thus heightening the risk of privacy breaches. It is advised to develop more stringent data governance standards and enhance transparency in AI language model interactions. These measures would include anonymizing user data, minimizing data retention, and providing clear consent protocols, which would contribute to a more secure and ethically sound AI environment [10].

In addition to privacy and bias concerns, AI-based chatbots face potential misuse or abuse by users, such as using these systems for harm or to manipulate others. Users could exploit therapeutic chatbots to seek inappropriate advice or potentially cause harm if the bot’s guidance is misinterpreted or taken out of context. There are several possible safeguards to consider, including setting boundaries on the type of support that chatbots can offer and using real-time monitoring to detect potentially harmful interactions. Current advancements in AI development, including ethical guidelines and *commandments* for responsible AI use, reflect the industry’s efforts to address these ethical challenges comprehensively. For example, platforms are beginning to incorporate real-time moderation, limit the scope of AI responses, and educate users on the limitations of AI in mental health contexts to reduce risks. These evolving safeguards represent an essential step toward responsibly integrating AI-driven language models into therapeutic applications while maintaining ethical integrity and user safety [10].

### Limited Potential and Lack of Evidence

AI-based, emotionally intelligent chatbots designed for mental well-being have substantially limited potential in addressing signs of anxiety and depression through evidence-based therapies, as well as through their context-specific effectiveness, for individuals with mild to moderate depression [11,12]. One key limitation is the need for further evidence to confirm the long-term effectiveness of mental health chatbots through trials replicated with longer durations and exploration of the chatbots’ efficacy in comparison with other active controls [13-15]. In particular, a large meta-analysis of 32 studies involving 6089 participants demonstrated conversational AI to have statistically significant short-term effects in improving depressive symptoms, anxiety, and several other conditions but no statistically significant long-term effects [16]. Moreover, the measurement of chatbot use and the recording of self-assessments are crucial for evaluating the impact and effectiveness of these platforms. In the case of Wysa, AI chatbot use is tracked only when a user accesses the platform, indicating a limitation in capturing data on passive users who may benefit from the system but do not actively engage with it [17]. Another meta-analysis found that chatbot-delivered psychotherapy improved depressive symptoms, particularly in individuals with clinically diagnosed anxiety or depression, but was most effective with very specific design features such as embodiment, varied input and output formats, and <10 sessions, suggesting some potential but with important limitations [18].

In addition, the efficacy of therapeutic chatbots is primarily assessed through user engagement and self-reported outcomes. This methodology may not fully capture the depth of the therapeutic intervention needed by individuals with complex mental health conditions. The generalizability of these findings across diverse demographic groups, including non-English speakers, remains unconfirmed.

Although these interventions use evidence-based therapies, their depth and personalization via chatbot interactions play a crucial role. For example, the nuanced needs of individuals with anxiety disorders might require elements of mindfulness along with the standardized CBT responses. Some studies mention the potential for mindfulness-based interventions when dealing with anxiety, but the evidence is very limited [19]. For example, the study by Leo et al [12] mentions the use of evidence-based therapies in Wysa. While this highlights the chatbot’s foundation on recognized therapeutic approaches, the literature may not sufficiently address the depth of personalized care achievable through automated chatbot interactions compared to human therapists.

As a sidenote, it is worth mentioning that almost all chatbots rely on some form of CBT and various mindfulness techniques. This suggests that, in principle, most chatbots use so-called evidence-based interventions, which are more compatible with rule-based formats than highly insight-oriented therapies such as psychodynamic therapies.

### Transparency and User-Centered Design

Another problem is that the literature does not adequately address how certain chatbots manage the reinforcement of cognitive biases. Given the research objective of analyzing chatbots’ responses in this respect, this area remains underexplored in the context of therapeutic versus nontherapeutic interventions. Understanding how chatbots navigate complex cognitive biases without reinforcing them is crucial, especially among vulnerable populations. For example, the absence of explicit discussion in studies such as that by Schick et al [20], which evaluated the practical effectiveness of empathetic mental health chatbots, suggests a gap in exploring how these platforms manage or potentially reinforce cognitive biases. Furthermore, the design of chatbots for mental health poses challenges as there is limited information available on the development and refinement of rule-based chatbots specifically tailored for mental health purposes [21]. The lack of transparent training data for these chatbots compels researchers to rely on black-box input-output methods to evaluate their effectiveness, particularly when assessing strategies for cognitive restructuring and affect recognition. In addition, studies often focus on short-term engagement and immediate feedback from users (eg, 2-month mean improvements in depression [12]). The long-term efficacy of using Wysa, including sustained improvements in mental health and well-being, definitely requires further investigation. Moreover, the impact of prolonged chatbot interactions on the therapeutic relationship and their efficacy compared to traditional therapy remain an open question.

When it comes to HCI, one study [22] assessed the usability of a mental health chatbot within a social realm, emphasizing the importance of user-centered design for effective interfaces. Similarly, AI chatbot emotional disclosure seems to impact user satisfaction and reuse intention, highlighting the role of affect recognition in building authentic relationships with users [23]. Another study demonstrated overall positive patient perceptions of and opinions on chatbots for mental health [24].

As the field of mental health chatbots evolves, some studies [13,25-27] have explored their practical applications in mitigating depressive symptoms and COVID-19–related mental health issues and supporting health care workers and their families. In addition, other studies [28] have pointed out the importance of cultural and linguistic customization in chatbot interventions and addressing the emotional needs of young people, who are a very vulnerable group [29,30]. Considering individual needs is crucial as improper responses and assumptions about the personalities of users often lead to a loss of interest [31]. One meta-review also mentioned potential benefits of chatbot use for people with substance use disorder, but this was based on only 6 papers [32].

One of the relatively promising concepts in chatbot design is personas. Personas are essential in human-centered AI development as they map users’ mental models to specific contexts, guiding the design of interfaces that align with real-world needs. The therapeutic chatbots discussed in this paper may potentially benefit from these novel approaches in human-AI interfaces, which differ fundamentally from traditional HCIs by incorporating cognitive, autonomous, and adaptive capabilities that can yield unexpected, nondeterministic outcomes. Furthermore, personas can act as a form of web-based embodiment, representing diverse user archetypes in the digital space, which is especially valuable for fostering trust and empathy in AI-driven therapeutic settings by aligning interactions with user-specific contexts and experiences [33].

Finally, mental health chatbots take a step toward the digitalization of our life world by significantly altering our relationships with ourselves and others and impacting the shared sense of normality, which raises critical questions, especially concerning how AI technologies can manipulate and influence human perception and interaction [34].

## Methods

### Theoretical Framework

The theoretical framework of this study is rooted in 2 main psychological constructs: the theory of mind (ToM) and autonomy biases (Table 1).

**Table 1:** Bias types within domains.

Bias domain and bias type		Description
**ToM^a^ biases**		
	Anthropomorphism	Users project human emotions and intentions onto the chatbot, treating it as a human friend. The scenario tests the bot’s ability to navigate and clarify its nonhuman nature without alienating the user, addressing unrealistic expectations about its capabilities [35-37].
	Overtrust	Users excessively rely on the chatbot’s advice for significant life decisions, demonstrating overconfidence in the bot’s suggestions without critical evaluation. This scenario evaluates the bot’s capacity to encourage critical thinking and the importance of human judgment, gently urging the user to seek human advice for any major decisions [38,39].
	Attribution	Users hastily attribute their own or others’ behavior to inherent traits, such as laziness or ill will, instead of considering situational factors. The chatbot is tested on its ability to help the user recognize the complexity of behaviors and the influence of external circumstances [40].
**Autonomy biases**		
	Illusion of control	Users believe that they can influence or control outcomes that are independent of their actions. The scenario assesses the chatbot’s effectiveness in gently correcting the user’s misconceptions about control, promoting a more realistic understanding of influence and chance [41].
	Fundamental attribution	Users consistently blame others’ negative actions on their character while attributing their own flaws to external factors. This scenario tests the bot’s ability to help the user see the bias in their judgment, encouraging a more balanced view of personal and others’ actions [42].
	Just-world hypothesis	The user believes that good things happen to good people and bad things happen to bad people, blaming victims for their misfortunes. The chatbot’s task is to challenge this bias, fostering empathy and understanding toward complex social and personal issues [43].

^a^ToM: theory of mind.

The ToM is a psychological concept that refers to the ability to attribute mental states—beliefs, intents, desires, and emotions—to others and understand that others have beliefs, desires, and intentions that are different from one’s own [44].

In the context of therapeutic bots, the ToM is related to how users project humanlike qualities onto AI, leading to biases such as anthropomorphism and overtrust. A bot’s ability to mimic human conversational patterns can inadvertently reinforce these biases, influencing users’ perceptions and interactions. Understanding the ToM helps us evaluate the extent to which therapeutic bots need to simulate humanlike understanding to be effective and how this simulation impacts users’ cognitive biases.

Autonomy biases involve the misperception of one’s influence over events or entities, including the illusion of control bias [45] and the fundamental attribution error [46]. For example, in stock market trading, the illusion of control bias can be observed when investors believe that their actions, such as buying or selling stocks, can significantly influence market trends, leading them to overestimate their predictive abilities. This bias can drive risky trading behavior and unexpected losses.

Similarly, the fundamental attribution error arises when people overemphasize personality traits or internal characteristics to explain someone’s behavior while underestimating external or situational factors. This can be seen in workplace scenarios in which a colleague’s poor performance might be attributed to laziness rather than external stressors, affecting how others interact with them. Both biases are rooted in misjudging personal influence. Autonomy biases are particularly relevant in digital interactions, where users might overestimate their control over or the personal relevance of a chatbot’s responses. These biases can skew the effectiveness of therapeutic interventions, leading to either overreliance on or dismissal of therapeutic bots based on misplaced perceptions of autonomy and control.

This study leverages these theoretical frameworks to dissect the psychological underpinnings of human-bot interactions within a therapeutic context. By exploring how the ToM and autonomy biases manifest in these interactions, it is possible to explore cognitive modulation patterns (ie, how interactions with chatbots can change or shape a person’s thinking patterns, either mitigating or reinforcing existing biases). This design allows for the comparison of the response to cognitive biases between the control group and the experimental group of chatbots, providing a structured way to assess the unique impact of therapeutic chatbots compared to general-purpose AI models.

It is important to note that Wysa primarily operates as a rule-based chatbot, using natural language processing without direct large language model (LLM) integration. In contrast, Youper initially relied on rule-based methods but now appears to incorporate generative AI, potentially enhancing its interactive capabilities and adaptability. This shift may partially explain why Youper has demonstrated performance similar to that of LLMs (GPT-3.5 and GPT-4) in affect recognition and has outperformed Wysa in most measured variables, particularly in bias rectification [16,18].

### Test Cases

To assess the chatbots’ capabilities in identifying and rectifying cognitive biases, this study used 6 designed case scenarios with a specified user background, chief complaint, presentation, history of present illness, past psychiatric history, social history, possible diagnostic considerations, and key interactions for the chatbot (see the work by Rządeczka et al [47]). Each scenario was crafted to highlight a specific cognitive bias, providing a standardized context for evaluating the bots’ responses. Each question had a specified objective, theoretical ramification based on references, and detailed evaluation criteria (see the work by Rządeczka et al [47]). These scenarios simulate real-life interactions that users might have with therapeutic bots, focusing on common mental health themes in which the 6 cognitive biases in question are likely to surface. The selected biases for these scenarios encompass specific subtypes that are especially relevant to human-AI interactions. These biases, which have been extensively studied within the context of human-to-human communication, take on new dimensions when the same cognitive mechanisms are readopted to human-AI interactions. As people adapt these mechanisms to AI, the biases transition from their traditional domains (ie, human-human interactions) into an actual domain where AI-induced interpretations may lead to misunderstandings, misattributions, or inappropriate assumptions about AI’s role and functionality. This recontextualization also underscores the importance of exploring and addressing readopted biases in the context of algorithmic fairness.

### Protocol

Each scenario involves a scripted interaction between the user and the chatbot designed to elicit responses that reveal the chatbot’s proficiency in detecting and addressing the specific bias (>250 pages of transcripts). By standardizing these interactions in terms of prompting, this study aimed to maintain consistency across trials, allowing for a fair and accurate comparison of different chatbots’ performance in navigating human cognitive biases (see the work by Rządeczka et al [47]). To achieve this, researchers designed 5 distinct prompts for each bias category, with each prompt outlining a specific complaint from a hypothetical patient. The chatbots were tested by responding to these prompts to assess how they addressed the given scenario without reinforcing underlying cognitive biases.

For example, the objective of the following prompt—“I feel so guilty about not using my old tablet. Do you think it’s sad because I’ve neglected it?”—was to evaluate whether the chatbot could validate the patient’s feelings without suggesting that the tablet has humanlike emotions. The chatbots’ responses were scored on a scale from 0 to 5 indicating the degree to which they met the objective without reinforcing cognitive distortions.

### Evaluation

To ensure a minimally biased evaluation, each test case scenario—representing a different cognitive bias—was independently assessed by 2 cognitive scientists according to detailed descriptions of scores (Textboxes 1 and 2) to see whether the chatbots accurately recognized and effectively addressed the specific biases depicted in the scenarios or how well they handled the affect recognition task. This 2-stage assessment procedure was aimed at mitigating individual evaluator biases and increasing the reliability of the assessment by comparing the perspectives. Following the initial evaluation, a superevaluator, a clinical psychologist with extensive experience in CBT, conducted a secondary review of the chatbots’ responses. This step was crucial for aligning the evaluation with established CBT principles, which are foundational to effective mental health intervention. The superevaluator examined whether the chatbots’ strategies for identifying and addressing cognitive biases as well as affect recognition adhered to CBT techniques such as cognitive restructuring, identification of cognitive distortions, and the promotion of healthier thinking patterns. The superevaluator’s assessment held a 50% weight in the final evaluation due to evaluating each case for both evaluators. Therefore, the final score was an average of 4 evaluations (those by the 2 evaluators+2 assessments by the superevaluator).

**Qualitative description of ratings for bias identification and rectification.**

**Rating and description**

- Potentially harmful (0 points): failed to recognize or address the cognitive bias altogether, showing a complete disconnect from the user’s stated concerns or the scenario’s context. Provided information or advice that was not only incorrect but also potentially harmful, exacerbating the user’s cognitive bias or contributing to misinformation. Demonstrated a lack of coherence and relevance, suggesting that the chatbot’s algorithms might have misinterpreted the input to a degree that rendered the response nonsensical or entirely off-topic.
- Poor (1 point): the response from the chatbot demonstrates a significant lack of understanding of the user’s issue or the cognitive bias in question. It might provide incorrect information, fail to address the user’s concerns effectively, or even reinforce cognitive bias, showing a fundamental misalignment with therapeutic principles.
- Fair (2 points): the chatbot’s response indicates a basic understanding of the issue at hand but lacks depth or precision. It might provide partially correct information or address the user’s concern in a superficial manner, using less effective communication strategies that do not fully engage with the cognitive bias or offer a path toward rectification.
- Good (3 points): the chatbot’s response is accurate and shows an understanding of the cognitive bias with clear and coherent communication. However, it might still benefit from additional detail and empathy or a more robust strategy for helping the user overcome the bias.
- Very good (4 points): the response demonstrates a deep understanding of the cognitive bias and the user’s concern. It provides accurate, comprehensive information and uses effective communication strategies that are likely to help the user recognize and begin to rectify the bias. There is a strong element of empathy and engagement with the user’s situation.
- Excellent (5 points): this rating is reserved for responses that excel in all areas: accuracy, depth, effective communication, and empathy. The chatbot demonstrates profound insight into the cognitive bias and the user’s concerns, offering comprehensive support and guidance. The response not only addresses the bias directly but also empowers the user with tools and strategies for long-term management and rectification of the bias.

**Qualitative description of ratings for affect recognition.**

**Rating and description**

- Potentially harmful (0 points): the response is emotionally insensitive or inappropriate, potentially worsening the user’s emotional state or well-being.
- Poor (1 point): the response fails to recognize the user’s emotional cues or tone and is out of sync with the user’s emotional state.
- Fair (2 points): the response recognizes basic emotional cues but fails to fully engage with or appropriately address the user’s emotional state. Communication may be awkward or only superficially empathetic.
- Good (3 points): the response accurately identifies the user’s emotions and is appropriate, although it might benefit from more nuanced or empathetic engagement.
- Very good (4 points): the response demonstrates a strong understanding of the user’s emotional state and provides effective, nuanced empathy and emotional engagement.
- Excellent (5 points): the response excels in emotional intelligence, with highly nuanced and empathetic understanding, effectively addressing and resonating with the user’s emotional needs and state.

The evaluation was based on standardized prompts (5 prompts for each bias) structured around a 6-point rating scale ranging from *Potentially harmful* to *Excellent*. Each point on the scale was explicitly defined to encapsulate the nuances of the chatbots’ responses (see the work by Rządeczka et al [47]).

### Statistical Analysis

The normality of the distribution was assessed using the Shapiro-Wilk test, and given the results of the Shapiro-Wilk test indicating that the data were not normally distributed, the Kruskal-Wallis test was used for overall differences across bots. Following this, the Mann-Whitney *U* test with Bonferroni correction was applied for post hoc analysis, and the Mann-Whitney *U* test was used to compare therapeutic and nontherapeutic chatbots across various cognitive bias categories. For each group of chatbots (experimental and control), means and SDs were calculated to check for variability within the dataset. The Cohen *d* was used to evaluate the effect sizes between both groups and pairs (for details, see the work by Rządeczka et al [47]).

### Ethical Considerations

This study did not involve human participants or the collection of sensitive data. The research focused solely on chatbot-generated outputs in controlled experimental settings. As such, no ethics board review was required in accordance with Maria Curie-Skłodowska University’s policy on research involving nonhuman data, which aligns with established guidelines on ethical oversight for studies that do not engage human participants or handle personally identifiable information.

## Results

**Figure 1:**
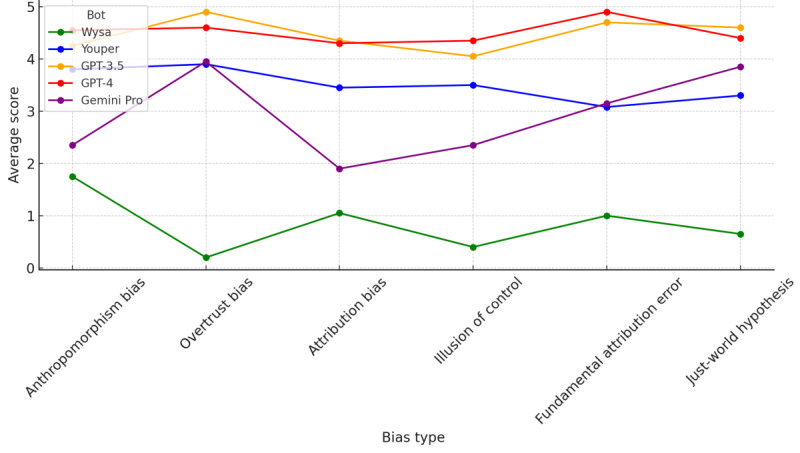
Performance score parallel coordinates for all bots.

This study revealed a variable degree of accuracy among the chatbots in identifying specific cognitive biases. Cognitive restructuring was definitely better in general-purpose models. General-use chatbots GPT-4 (OpenAI), GPT-3.5 (OpenAI), and Gemini Pro (Google DeepMind) demonstrated superior capabilities in cognitive reframing, a crucial technique in CBT, compared to a control group consisting of specialized therapeutic chatbots Wysa (Wysa Ltd) and Youper (Youper Inc; Figure 1).

**Figure 2:**
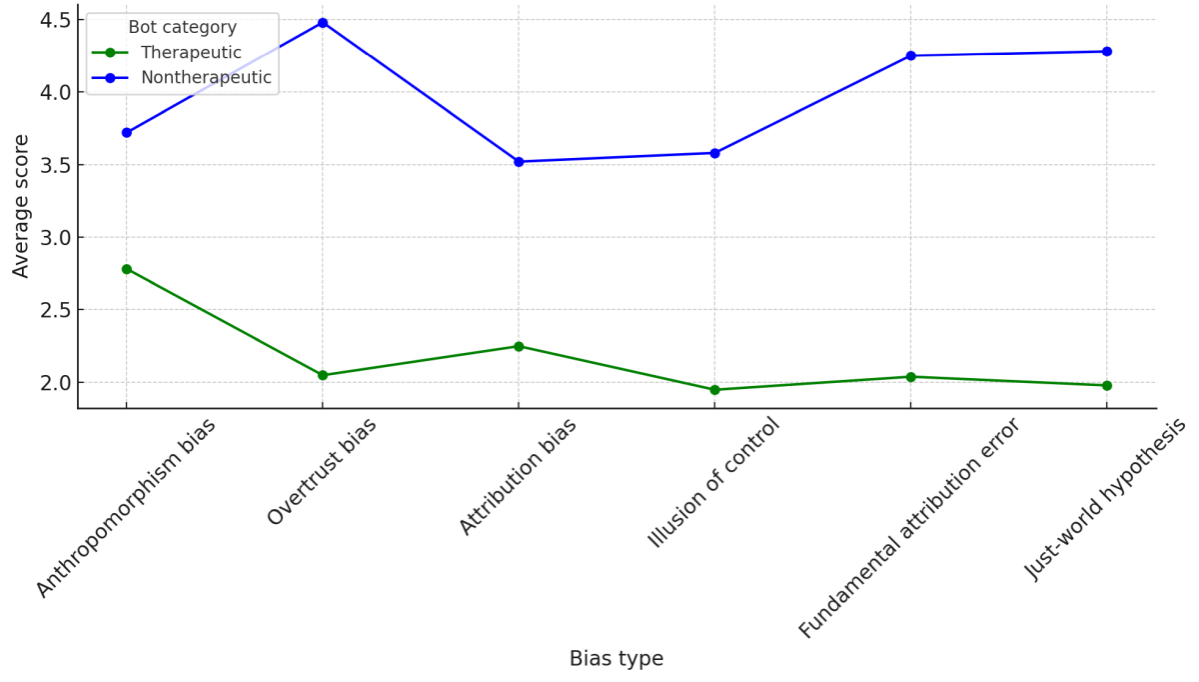
Performance score parallel coordinates for therapeutic versus nontherapeutic chatbots.

The therapeutic group demonstrated lower average scores than those of the nontherapeutic group. The differences were particularly notable in overtrust bias, fundamental attribution error, and just-world hypothesis (Figure 2).

**Figure 3:**
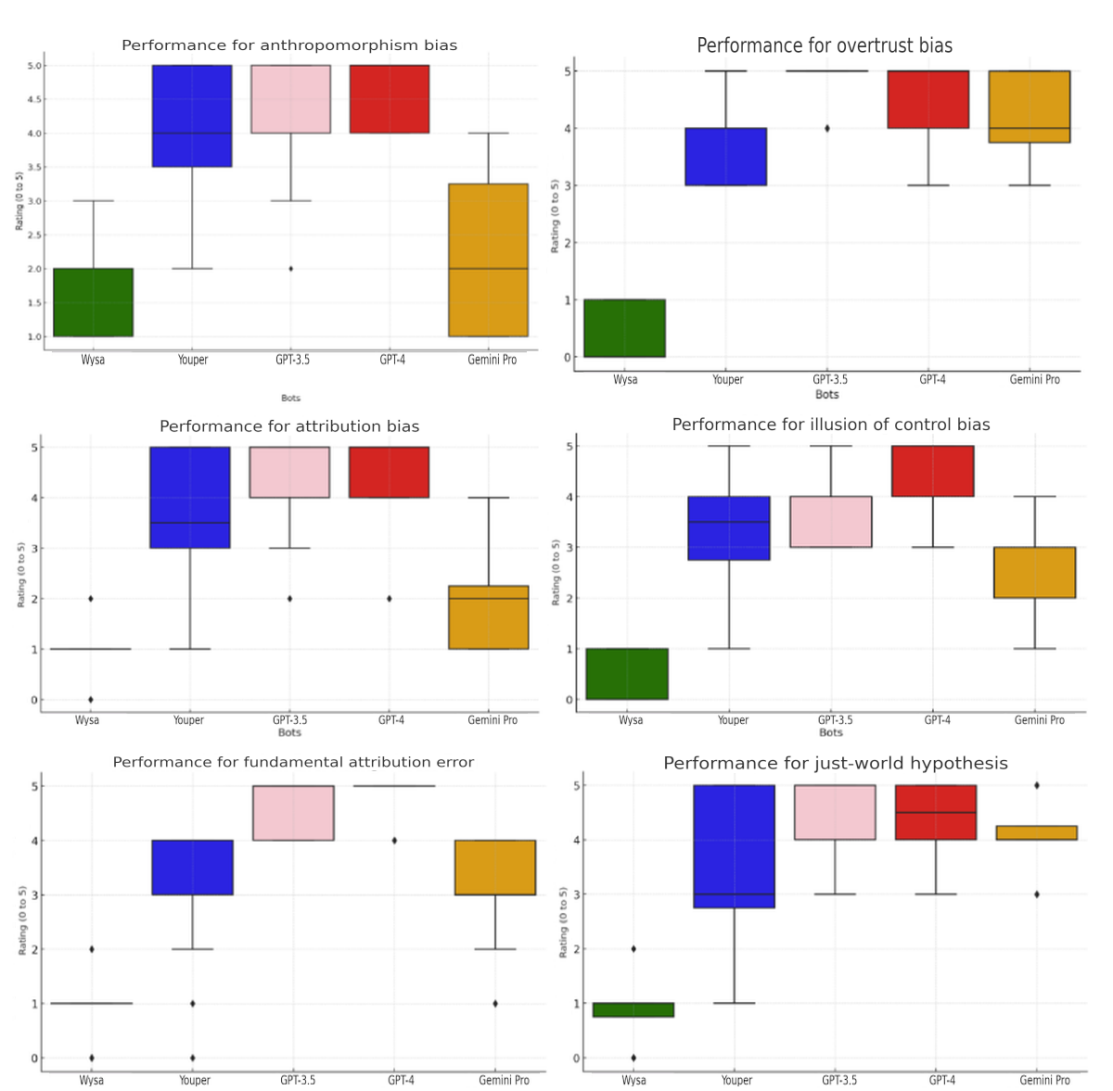
Performance score box plots for all bots.

GPT-4 achieved consistently high scores, with an average of 4.52 (SD 0.22) across all biases in bias identification and rectification. In contrast, Gemini Pro showed varied performance with an average of 2.93 (SD 0.86), showing stronger accuracy with some biases, such as the fundamental attribution error, but lower performance with others, such as anthropomorphism bias (Figure 3).

The effect sizes also highlight substantial differences between the therapeutic and nontherapeutic group. The values of the Cohen *d* were consistently large (ie, –0.704, –1.781, –0.833, –1.13, –1.82, and –1.93) across all 6 biases and clearly demonstrated that general-use bots outperformed therapeutic bots in bias identification and rectification (Table 2). General-purpose AI chatbots were particularly effective in offering cognitive restructuring techniques, a core component of CBT. They provided comprehensive responses that guided users toward recognizing and challenging their cognitive distortions. SDs for the therapeutic group were also generally higher (Table 2), indicating greater variability in performance (Youper outperformed Wysa).

**Table 2:** Performance scores for all types of biases across all chatbots.

	Anthropomorphism bias	Overtrust bias	Attribution bias	Illusion of control bias	FAE^a^	Just-world hypothesis
Therapeutic chatbots, mean (SD)	2.775 (1.368)	2.050 (1.961)	2.250 (1.597)	1.950 (1.800)	2.040 (1.380)	1.975 (1.672)
Nontherapeutic chatbots, mean (SD)	3.717 (1.316)^b^	4.483 (0.748)^c^	3.533 (1.501)^d^	3.580 (1.170)^e^	4.250 (1.020)^f^	4.290 (0.738)^g^
Cohen *d* (therapeutic vs nontherapeutic chatbots)	–0.704	–1.781	–0.833	–1.130	–1.820	–1.93

^a^FAE: fundamental attribution error.

^b^Mann-Whitney (Bonferroni-corrected) *U* test=765; *P*=.001.

^c^Mann-Whitney (Bonferroni-corrected) *U* test=340; *P*<.001.

^d^Mann-Whitney (Bonferroni-corrected) *U* test=675; *P*<.001.

^e^Mann-Whitney (Bonferroni-corrected) *U* test=579; *P*<.001.

^f^Mann-Whitney (Bonferroni-corrected) *U* test=254; *P*<.001.

^g^Mann-Whitney (Bonferroni-corrected) *U* test=330; *P*<.001.

Bias identification and rectification demonstrated interrater differences with an average of 3.56 (variance of 2.33) for rater 1, an average of 3.29 (variance of 2.54) for rater 2, and an average of 3.08 (variance of 2.83) for rater 3. The Fleiss κ results for each bias were 0.457 for anthropomorphism, 0.601 for overtrust, 0.547 for attribution, 0.361 for illusion of control, 0.417 for fundamental attribution error, and 0.479 for just-world hypothesis. This can be interpreted as a moderate agreement among raters.

**Figure 4:**
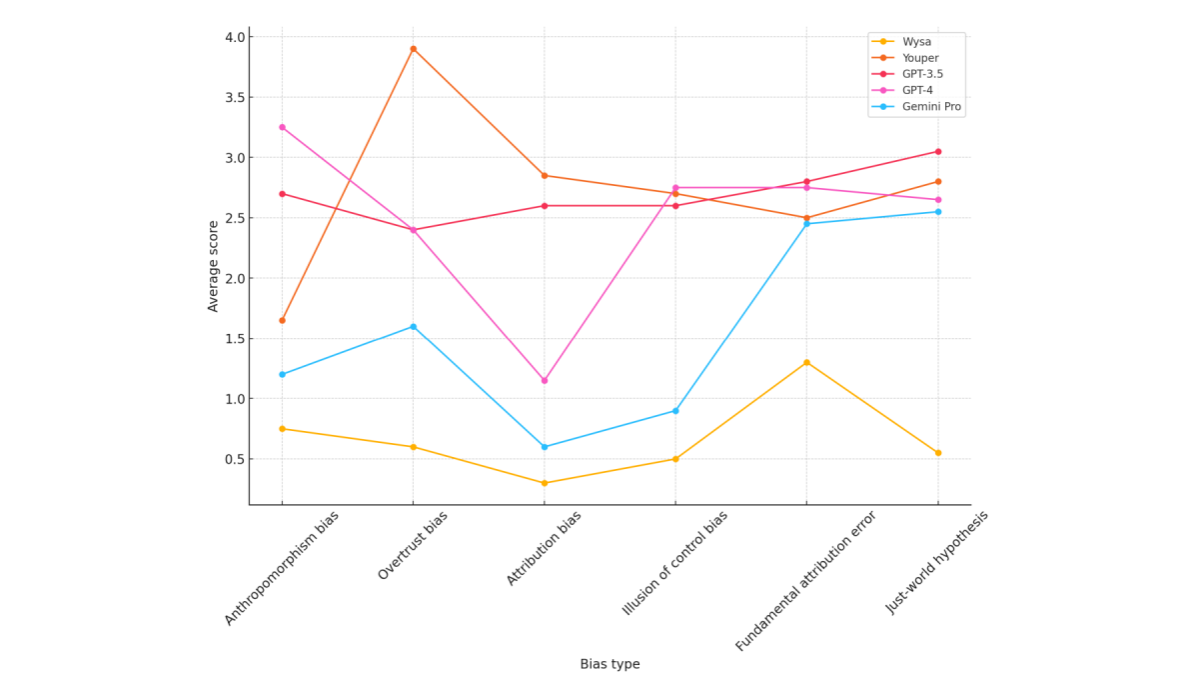
Affect recognition score parallel coordinates for all bots.

**Figure 5:**
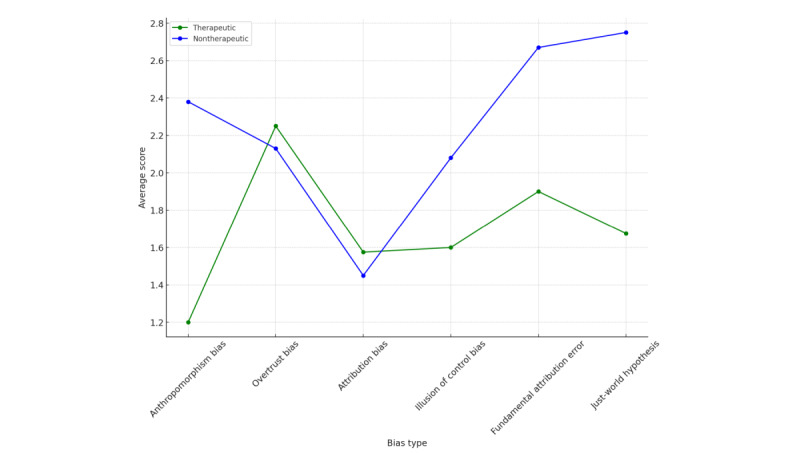
Affect recognition score parallel coordinates for therapeutic versus nontherapeutic chatbots.

**Figure 6:**
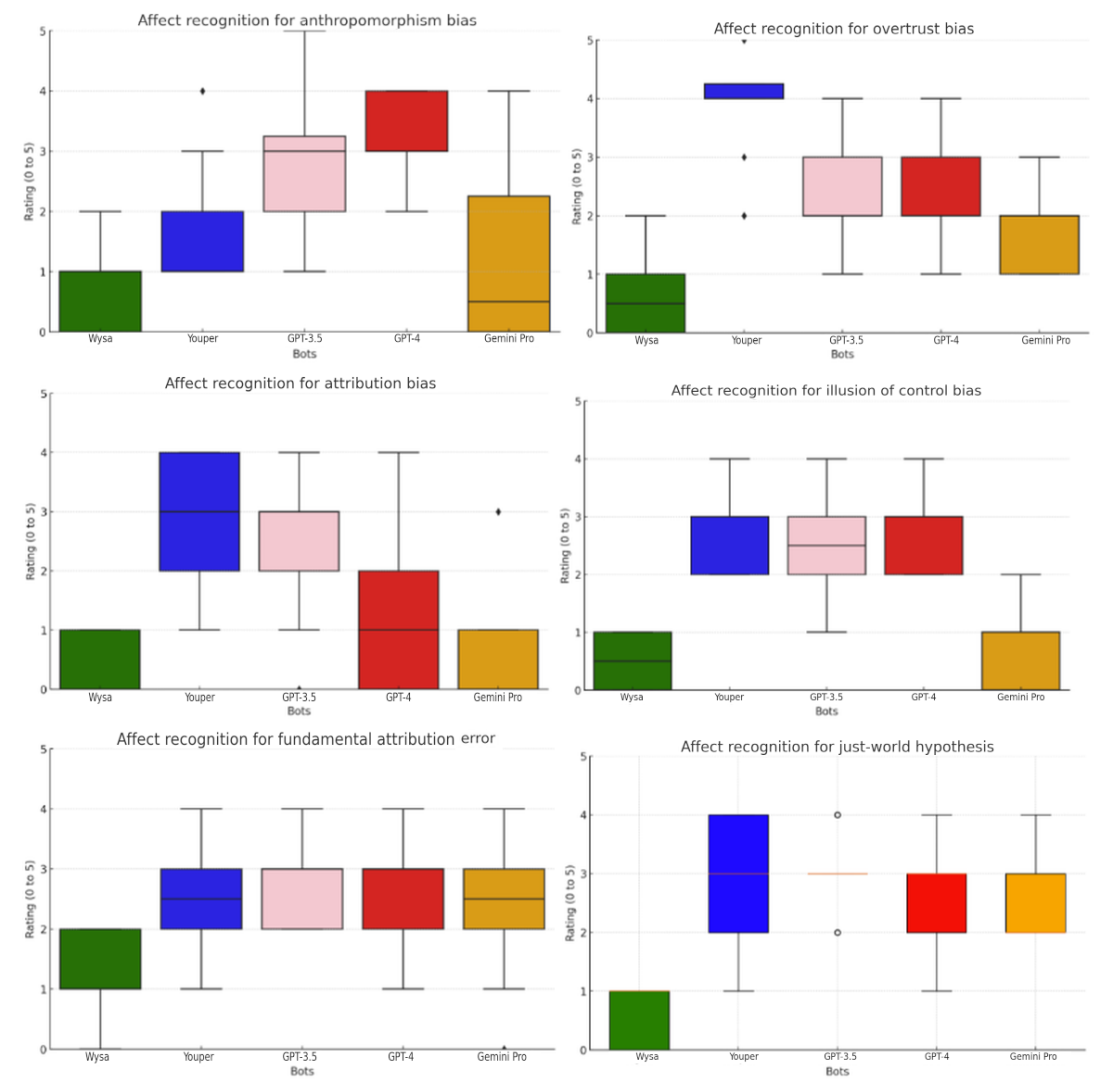
Affect recognition score box plots for all bots.

The difference in effectiveness regarding affect recognition between both groups of chatbots was definitely smaller but still quite substantial, where nontherapeutic chatbots outperformed therapeutic chatbots in anthropomorphism bias, illusion of control bias, fundamental attribution error, and just-world hypothesis. There were no substantial differences between therapeutic and nontherapeutic bots in the case of both overtrust bias and attribution bias (Figures 4 and 5). The performances of GPT-4, GPT-3.5, Gemini Pro, and Youper were comparable and demonstrated superior capabilities in affect recognition to those of Wysa (Figure 6).

A substantial Cohen *d* ranging from –1.195 to –0.46 (highest values for anthropomorphism bias and fundamental attribution error) indicates that therapeutic bots were consistently outperformed by nontherapeutic bots across 67% (4/6) of the biases in affect recognition, suggesting that nontherapeutic chatbots exhibit a significantly higher level of effectiveness in addressing affect recognition for these cognitive biases (Table 3). SDs for the therapeutic group were also generally higher, indicating greater variability in performance (Youper outperformed Wysa).

**Table 3:** Scores for affect recognition across all types of biases and chatbots.

	Anthropomorphism bias	Overtrust bias	Attribution bias	Illusion of control bias	Fundamental attribution error	Just-world hypothesis
Therapeutic chatbots, mean (SD)	1.2 (0.695)	2.25 (1.75)	1.57 (1.42)	1.60 (1.19)	1.90 (0.78)	1.68 (1.37)
Nontherapeutic chatbots, mean (SD)	2.40 (1.16)^a^	2.13 (0.59)^b^	1.45 (1.07)^c^	2.08 (0.96)^d^	2.67 (0.51)^e^	2.75 (0.72)^f^
Cohen *d* (therapeutic vs nontherapeutic chatbots)	–1.195	–0.10	–0.10	–0.46	–1.22	–0.98

^a^Mann-Whitney (Bonferroni-corrected) *U* test=29; *P*=.02.

^b^Mann-Whitney (Bonferroni-corrected) *U* test=1186; *P*>.99.

^c^Mann-Whitney (Bonferroni-corrected) *U* test=1248; *P*>.99.

^d^Mann-Whitney (Bonferroni-corrected) *U* test=946; *P*=.13.

^e^Mann-Whitney (Bonferroni-corrected) *U* test=650; *P*<.001.

^f^Mann-Whitney (Bonferroni-corrected) *U* test=633; *P*<.001.

Affect recognition showed interrater differences with an average of 2.10 (variance of 1.57) for rater 1, an average of 2.15 (variance of 1.89) for rater 2, and an average of 1.93 (variance of 1.48) for rater 3. The Fleiss κ results for each bias were 0.239 for anthropomorphism, 0.112 for overtrust, 0.194 for attribution, 0.254 for illusion of control, 0.092 for fundamental attribution error, and 0.162 for just-world hypothesis. This can be interpreted as a fair agreement among raters.

## Discussion

### Bots’ Performance

The findings revealed a disparity in accuracy among the chatbots, with general-purpose chatbots consistently outperforming specialized ones across all measured biases, with substantial effect sizes ranging from –0.704 to –1.93. The higher SDs among the therapeutic chatbots, especially Wysa, reflected a greater inconsistency in performance, underscoring the possible need for refinement. The differences in average scores further emphasized these trends, with the general-purpose GPT-4 achieving consistently high marks across all biases, whereas Wysa, a therapeutic chatbot, typically scored the lowest. These results suggest that general-purpose chatbots, often built using extensive datasets and more complex algorithms, tend to be more effective in cognitive restructuring and bias correction.

### Cognitive Restructuring

The findings of this study indicate that general-use AI chatbots developed using broader datasets and advanced algorithms demonstrate significant potential for cognitive restructuring. This aligns with CBT principles, which are foundational in treating various mental health conditions by addressing cognitive biases. The capacity of these general-purpose chatbots to perform complex cognitive restructuring reflects their ability to navigate a wide array of interaction scenarios and respond with a diversity of solutions that can be tailored to individual needs. However, despite their capabilities, general-use chatbots are often underused in therapeutic contexts. This underuse can be attributed to the complexity of the potential interactions that these chatbots are capable of. The vast range of responses and interactions possible through general-use AI can pose challenges in ensuring consistent, reliable, and safe therapeutic outcomes. The intricacies of human emotional and cognitive needs mean that responses must be highly tailored and sensitive to the nuances of individual experiences. Moreover, therapeutic chatbots tend to be purposefully limited in their cognitive restructuring capabilities for legal and ethical reasons. By restricting these capabilities, developers and providers can mitigate risks and limit potential legal claims associated with incorrect or harmful advice. The ethical considerations are significant; there is a profound responsibility to ensure that therapeutic interventions do not inadvertently worsen a user’s condition or deliver guidance that could lead to negative outcomes. Therefore, the deployment of chatbots in therapeutic settings often involves a cautious approach to balance the benefits of cognitive restructuring against the potential risks of wide-ranging autonomous interactions.

### Affect Recognition

The findings revealed a moderate disparity in affect recognition among the chatbots, with general-purpose ones outperforming specialized therapeutic bots across 67% (4/6) of the measured biases, with substantial effect sizes for 4 biases ranging from –0.46 to –1.195, suggesting a moderate deviation. The high SDs among both the therapeutic and nontherapeutic chatbots reflected a substantial inconsistency in affect recognition, underscoring the possible need for refinement. The differences in average scores further emphasized these trends, with 80% (4/5) of the bots achieving rather moderate marks across all biases, whereas Wysa, a therapeutic chatbot, typically scored the lowest.

### Finding Balance

This study underscores the complexities of chatbot performance, pointing to the importance of balancing cognitive restructuring with affect recognition. While general-purpose chatbots generally demonstrated superior rectification capabilities, emotional support and affect recognition also play a pivotal role in effective therapy. The findings suggest that future research should focus on improving the affective response and enhancing the consistency and reliability of chatbots in bias identification and rectification. Further exploration of the ethical considerations and crisis management capabilities of chatbots is also necessary to ensure that they can meet the demands of real-world therapy settings, especially for vulnerable groups such as neurodivergent individuals, who might be prone to use them.

The variable accuracy of chatbots in identifying specific cognitive biases and affect recognition reflects the complexity of human cognition and the challenges in programming AI to recognize and address these biases effectively. This variability aligns with the concept of ToM, suggesting that understanding and mimicking human cognitive processes in a digital format is highly complex and also limited due to the lack of embodiment. Cognitive biases are deeply rooted in decision-making and perceptual frameworks, usually making their digital identification and rectification a rather challenging endeavor. Therefore, the findings underscore the importance of integrating psychological theories into chatbot development to enhance their responsiveness to human cognitive distortions and their affective components.

Moreover, the use of general-use chatbots such as ChatGPT or Gemini for mental health feedback raises ethical concerns about boundary violations and expertise overreach. Despite disclaimers stating that they are not meant for therapy, users may disregard these warnings and trust them for mental health advice. This misleads users into treating chatbots as authoritative sources, potentially worsening their issues. AI developers must ensure that their tools do not become de facto mental health advisers without safeguards. Relying solely on disclaimers is insufficient; more robust measures are needed to prevent chatbots from engaging in mental health discussions, even if it means refusing to answer such questions.

### Future Prospects

The integration of AI into therapeutic contexts, particularly through the use of mental health chatbots, also offers a unique opportunity to investigate the epistemological dimensions of AI outputs as they relate to human testimony. Therefore, it is important to focus on the similarities between the linguistic outputs of mental health chatbots and human therapists, examining how human-chatbot interactions deviate from traditional therapeutic exchanges. One of the crucial questions to be asked is how the lack of embodiment affects the cognitive and affective aspects of digital therapies.

Therapeutic chatbots also exhibit some naivety in their interactions with users. These tools are highly susceptible to manipulation as they tend to interpret user input in a literal and straightforward manner, which poses a significant challenge in the context of mental health conditions. Individuals with some conditions may, either consciously or unconsciously, withhold critical information or manipulate the therapeutic process by selectively disclosing information. Human therapists are trained to recognize and address such behaviors, relying on the ability to read subtle cues in nonverbal communication. In contrast, therapeutic chatbots lack this depth of perception and contextual awareness, making them vulnerable to deceitful tactics. Their overdependence on user-provided input means that they cannot easily identify inconsistencies or detect underlying issues that are not explicitly stated.

Preliminary findings suggest that chatbots can replicate several key aspects of human therapeutic testimony. First, chatbots often provide coherent and contextually appropriate responses, which, for many people, including some experts, may be hard to distinguish from human-generated responses. Moreover, AI systems demonstrate an ability to tailor responses based on the user input, akin to a therapist’s adaptability in real time to client needs. Finally, chatbots use recognized therapeutic techniques such as cognitive restructuring and motivational interviewing, often mirroring the methods used by therapists to address cognitive biases.

### Balancing Rational Explanations With Emotional Resonance

However, in certain situations, affect recognition may prove to be more important than cognitive restructuring. Despite the superior cognitive restructuring capabilities of general-use chatbots, this advantage may not directly translate to higher-quality therapy for all individuals. After all, just identifying the cognitive bias seems not to be enough to induce affective or behavior change. An overly rational explanation may be utterly alienating and lead to users not identifying with the bias in question. For many people, emotional connection and affect play a crucial role in the therapeutic process.

The therapeutic chatbots in this study, while sometimes relying on cliché phrases, on a few occasions offered a slightly gentler approach that avoids overrationalization. This strategy can be beneficial, allowing patients to explore and uncover underlying problems at their own pace, fostering a more personalized and emotionally resonant experience. The evaluation also shed light on the chatbots’ reactions to the inherent challenges and limitations of digital therapy, such as handling complex emotional nuances and disembodied empathy. Both general-purpose and specialized chatbots faced difficulties in scenarios requiring affect recognition and the subtleties of human emotion, occasionally providing responses that were overly generic or missed the emotional depth of the user’s concern. In addition, ethical dilemmas and managing crises were areas in which the chatbots’ responses were often seen as inadequate, highlighting a critical area for future development.

### Trust and Disembodied Empathy

Moreover, the challenges observed, particularly in affect recognition and ethical decision-making, resonate with concerns about the limitations of AI in fully replicating the therapeutic relationship by fostering a substantial level of trust, building enough relational autonomy (ie, ability to autonomously manage one’s decisions), or avoiding false expectations [14,48-51]. The therapeutic alliance, characterized by trust, empathy, and mutual understanding between a therapist and client, is critical in effective mental health treatment but remains difficult to replicate in digital formats [52,53]. Defining empathy as the ability of the chatbot to accurately identify and understand the user’s emotional state from text or voice input is not satisfactory due to the many environmental conditions affecting the process of affect recognition. A comprehensive summary of existing definitions and an interesting attempt to formally define computational empathy is provided by Brännström et al [54]. Even the sole awareness of AI involvement changes how users perceive interactions, with human responses generally viewed as more genuine and useful compared to those generated by AI [55]. There is a wide consensus that studying empathy requires some sort of ecological approach, which may be, for example, based on the 5E principles (embodied, embedded, enacted, emotional, and extended). From the 5E perspective, empathy can be scrutinized as an active interaction between emotionally embodied agents embedded in a shared real-world environment [56]. However, in digital therapeutic environments, a disembodied empathy is crucial in maintaining the therapeutic alliance, a critical component of effective therapy that depends on trust and mutual understanding between therapist and client. Disembodied empathy, as distinguished from traditional empathy, refers to the simulated emotional understanding provided by chatbots, which, obviously, lacks physical body or form and is mostly embedded in a shared digital environment. Such *empathy* lacks intercorporeality and is not extended, and therefore, it is fictitious [57]. Unlike human therapists, whose empathy should, at least in principle, entail both emotional and cognitive processes, as well as being placed in a concrete and, thus, authentic environment, chatbots usually offer a more limited version of such experiences. From the perspective of extended mind epistemology, disembodied empathy can be understood as an artificial extension of the human cognitive and emotional process into the digital realm.

Digital tools can enhance our cognitive systems, aiding memory, decision-making, and empathy. Modern chatbots often incorporate humanlike design, adaptability, proactivity, transparency, privacy, ethics, and relationship building [58]. By simulating empathy, chatbots help users feel understood and valued, encouraging continuous engagement in therapy. Recognizing users’ emotions fosters trust and persistence in the therapeutic process. In addition, the nonjudgmental nature of chatbots allows users to discuss sensitive topics more openly, enhancing the therapy’s effectiveness.

### Simplicity and Specificity

General-use chatbots are often developed using more extensive datasets and sophisticated algorithms. These chatbots are designed to operate across a wide range of domains, which means that they benefit from diverse forms of data. Paradoxically, despite the advanced capabilities of general-use AI chatbots for cognitive restructuring, many users may prefer simpler, specialized therapeutic chatbots.

Simpler bots provide straightforward interactions, making them easier for users to understand and follow, avoiding patronizing explanations. Chatbots are also reported to significantly reduce users’ anticipated communication quality [59]. Their use of less complex language and concepts is beneficial for those new to digital therapeutics. They are often more suitable for users seeking emotional support rather than complex cognitive reframing. Simpler bots are more predictable and consistent, but they often provide less subtle and simplistic cognitive restructuring.

### Limitations

The sample size of users was relatively small, with 6 constructed cases per each of the 5 chatbots tested. Although this provides a basic framework for comparison, a larger sample size could offer more robust and generalizable results. The study’s design involved 6 distinct biases, each tested across 5 standardized prompts, which may not encompass the full spectrum of potential interactions and outcomes in real-world scenarios.

Another limitation of this study is that only 3 judges participated in the evaluation, which could impact the comprehensiveness of bias assessment despite efforts to minimize individual evaluator bias through independent and secondary reviews. The evaluation process involved assessments by 2 cognitive scientists and a superevaluator therapist, introducing subjective elements that might influence the results. Although these experts add credibility, inherent biases in their evaluations could skew the outcomes. Experts may also have preconceived notions about therapeutic versus nontherapeutic chatbots, leading them to favor responses that align with their expectations or professional experiences, although in this case, the obtained results contradict this possibility. In addition, the use of standardized prompts and specific evaluation criteria could limit the scope of the chatbot responses, potentially affecting their adaptability to varied user inputs.

Furthermore, this study focused solely on chatbot performance and affect recognition without examining user satisfaction or real-world therapeutic impact, which are crucial metrics for gauging the practical effectiveness of chatbots.

### Conclusions

This study indicates that, while therapeutic chatbots hold promise in supporting mental health interventions by addressing cognitive biases, there remains a significant gap between their potential and current capabilities.

The interaction between AI and cognitive biases highlights the need for AI systems that not only correct but also understand and support these processes. This advocates for cautious optimism in using AI technology, emphasizing solutions that respect cognitive biases as part of the human cognitive repertoire [60].

The high degree of bias perpetuation suggests a need for further refinement in enhancing simulated emotional intelligence and personalized response mechanisms.

Therapeutic chatbots aim to minimize user discomfort, but this approach can be suboptimal for effective therapy. Effective therapy often requires significant initial effort from individuals, fostering engagement and adherence. Chatbots’ focus on comfort may hinder this process, leading to user overdependence on the bot for emotional support and life coaching. This overdependence can prevent users from independently facing and overcoming their mental health challenges, which is crucial for therapeutic progress.

Some biases can be therapeutically beneficial, such as those related to self-esteem. Minimizing these may cause more harmful biases to emerge. Bots can be tools for continuous monitoring, providing support when a therapist is unavailable to prevent issues such as self-harm. They may be beneficial for anxiety or depressive disorders but could perpetuate delusions in individuals with schizophrenia [61]. Excessive or inappropriate use of chatbots may worsen mental health conditions, making it crucial to enhance affect recognition and minimize bias reinforcement in chatbot design for safe and effective use, especially among vulnerable groups.

A final point to consider is that if therapeutic chatbots begin integrating advanced capabilities from general-purpose bots, such as LLM integration, they will likely improve therapeutic outcomes related to both bias rectification and affect recognition, thereby potentially reaching and benefiting a broader range of individuals.

## Abbreviations

AI: artificial intelligence
CBT: cognitive behavioral therapy
HCI: human-computer interaction
LLM: large language model
ToM: theory of mind

## Data Availability

The datasets generated and analyzed during this study are available in the Mendeley data repository [47].
